# The Heating Efficiency and Imaging Performance of Magnesium Iron Oxide@tetramethyl Ammonium Hydroxide Nanoparticles for Biomedical Applications

**DOI:** 10.3390/nano11051096

**Published:** 2021-04-23

**Authors:** Mohamed S. A. Darwish, Hohyeon Kim, Minh Phu Bui, Tuan-Anh Le, Hwangjae Lee, Chiseon Ryu, Jae Young Lee, Jungwon Yoon

**Affiliations:** 1 School of Integrated Technology, Gwangju Institute of Science and Technology, Gwangju 61005, Korea; msa.darwish@gmail.com (M.S.A.D.); duplisona@gm.gist.ac.kr (H.K.); buiminhphu@gist.ac.kr (M.P.B.); tuananhle@gist.ac.kr (T.-A.L.); 2 Egyptian Petroleum Research Institute, 1 Ahmed El-Zomor Street, El Zohour Region, Nasr City, Cairo 11727, Egypt; 3 School of Materials Science and Engineering, Gwangju Institute of Science and Technology, Gwangju 500-712, Korea; hj31p@gist.ac.kr (H.L.); ryuchiseon@gist.ac.kr (C.R.)

**Keywords:** hyperthermia, magnesium iron oxide, magnetic particle imaging, nanoparticle

## Abstract

Multifunctional magnetic nanomaterials displaying high specific loss power (SLP) and high imaging sensitivity with good spatial resolution are highly desired in image-guided cancer therapy. Currently, commercial nanoparticles do not sufficiently provide such multifunctionality. For example, Resovist^®^ has good image resolution but with a low SLP, whereas BNF^®^ has a high SLP value with very low image resolution. In this study, hydrophilic magnesium iron oxide@tetramethyl ammonium hydroxide nanoparticles were prepared in two steps. First, hydrophobic magnesium iron oxide nanoparticles were fabricated using a thermal decomposition technique, followed by coating with tetramethyl ammonium hydroxide. The synthesized nanoparticles were characterized using XRD, DLS, TEM, zeta potential, UV-Vis spectroscopy, and VSM. The hyperthermia and imaging properties of the prepared nanoparticles were investigated and compared to the commercial nanoparticles. One-dimensional magnetic particle imaging indicated the good imaging resolution of our nanoparticles. Under the application of a magnetic field of frequency 614.4 kHz and strength 9.5 kA/m, nanoparticles generated heat with an SLP of 216.18 W/g, which is much higher than that of BNF (14 W/g). Thus, the prepared nanoparticles show promise as a novel dual-functional magnetic nanomaterial, enabling both high performance for hyperthermia and imaging functionality for diagnostic and therapeutic processes.

## 1. Introduction

Iron oxide nanoparticles (IONPs) are widely used in magnetic separation, drug-delivery, imaging, and hyperthermia cancer treatments, due to their biocompatibility, magnetic-imaging capability, and hypothermic characteristics [1,2,3,4]. In particular, one of the most developed IONP techniques is magnetic hyperthermia in which heat is generated under an alternating magnetic field (AMF) to destroy tumor cells. IONPs that can elevate the temperature of the target tissue above 45 °C are suitable for tumor treatment [4]. For effective treatment, the cancerous tissue temperature should reach 42 to 45 °C, whereas temperatures over 50 °C destroy the cancer cells via thermoablation.

A short treatment time is highly desirable for safe and effective hyperthermia treatment. The specific loss power (SLP) is used as a parameter to investigate how much energy is absorbed per magnetic nanoparticles mass (NPs) under AMF [2,3]. Hence, extensive efforts have been made to produce magnetic nanomaterials possessing high SLP values. Concurrently, there is increasing interest in using NPs as imaging agents to monitor the location and distribution of nanomaterials precisely and stably for magnetic-particle imaging (MPI) applications [5,6,7].

MPI detects the positions and concentrations of IONPs via time-varying magnetic fields. Thus, the IONPs act as MPI tracer agents, because they can be magnetized instantaneously by an external magnetic field and can stimulate a nonlinear response in a near-zero magnetic field [6]. Compared with existing imaging modalities, MPI is recognized as a favorable tool for cancer diagnosis, as it offers the advantages of zero background signal, zero signal reduction with increasing tissue depth, quantitative linearity [7], and high sensitivity. Additionally, for MPI, there is no need for ionizing radiation.

Various techniques have been developed for IONP fabrication, including co-precipitation, thermal decomposition, and hydro-/solvothermal processes [8,9,10,11,12,13]. Among them, the thermal decomposition technique has been widely studied. Thermal decomposition allows for fine control over the morphology and size of IONPs by changing the synthetic parameters (e.g., precursors, surfactants, pH value, and reaction time) [12,13]. The surface properties of IONPs critically influence the overall performance of the materials. Hence, surface modification of IONPs is usually carried out either during or after synthesis [9,10].

The presence of coatings on the surface of IONPs can protect the magnetic core from oxidative environments, enhance stability and compatibility, and improve tumor-targeting efficiency. Generally, IONPs are biocompatible and non-toxic; thus, they are suitable for biomedical applications. It should be noted that biocompatibility is also strongly influenced by the coating material [14,15,16,17]. To date, two types of iron oxide NPs have been applied in clinical treatment: (i) shelled with polysaccharide layer and (ii) shelled with silica layer [14].

The current challenge in applying IONPs for diagnostic and therapeutic processes is the development of a smart theranostic agent with high performance for hyperthermia treatment and MPI. Currently, RES NPs is promising as an MPI contrast agent; however, it has a low SLP value that restricts its use in therapeutic hyperthermia cancer treatments. On the other hand, BNF has a relatively high SLP value in hyperthermia systems, but a low MPI sensitivity. Therefore, IONPs that can combine, in a single material, the unique advantages of commercially and clinically used IONPs will be promising theranostic agents. As ideal theranostic agents, magnetic IONPs should be non-toxic and biocompatible with a high magnetization value.

Controlling the size and morphology of NPs is an effective technique for improving SLP, image resolution, and biocompatibility [5,6,7]. Bauer et al. prepared zinc-doped magnetite cubic NPs and achieved good MPI performance and a high SLP of 1019.2 W/g under the action of an alternating magnetic field of strength 16 kA/m and frequency 380 kHz; however, these magnetic-field parameters exceed the safety limit of SLP testing, i.e., 5 × 10^9^ A m^−1^s^−1^ [5]. Recently, Dadfar et al. prepared citrate-coated superparamagnetic IONPs and achieved good MPI performance and a high SLP of 350 W/g, with a magnetic field of strength 46 kA/m and frequency 186 kHz; however, the main limitation of the study was also that the magnetic field parameters exceeded the safety limit [6]. In another report, Song et al. prepared carbon-coated FeCo NPs and achieved good MPI performance and a high SLP of 406 W/g with a magnetic field of strength 100 kA/m and frequency 30 kHz [7]; however, they did not clearly address the potential biocompatibility issue, as the dissolution of the FeCo NPs would lead to the release of toxic Co^2+^ ions inside the living organism [17].

In this study, we synthesized and characterized high-performance nanomaterials that have high SLP and high sensitivity with good spatial resolution, based on magnesium iron oxide@tetramethyl ammonium hydroxide (MgIONPs@TMAH) NPs. To our knowledge, the efficiency of MgIONPs@TMAH as a theranostic agent has not been investigated. In this study, we focused on the dual features of high SLP performance and high MPI sensitivity necessary for the application of MgIONPs@TMAH in practice. To this end, MgIONPs@TMAH nanoparticles were compared to the commercially and clinically used IONPs.

## 2. Experimental Work

### 2.1. Materials

Iron (III) acetylacetonate (97% purity), magnesium acetate tetrahydrate (≥99% purity), benzyl ether (98% purity), tetramethyl ammonium hydroxide (10 wt% in H_2_O) and oleic acid (≥99% purity) were purchased from Sigma Aldrich (St. Louis, MO, USA). Commercial BNF NPs (iron oxide nanoparticles coated with dextran (Micromod Partikeltechnologie GmbH, Rostock, Germany) and Ferucarbotran (RES NPs) iron oxide NPs shelled with carboxydextran (Meito Sangyo Co., Ltd., Nagoya, Japan)) were used in the study.

### 2.2. Synthesis of Magnesium Iron Oxide@Tetramethyl Ammonium Hydroxide Nanoparticles (MgIONPs@TMAH)

MgIONPs@TMAH nanoparticles were prepared by the following two steps consisting of the synthesis of hydrophobic MgIONPs and surface modification with TMAH, as described below.

#### 2.2.1. Preparation of Hydrophobic Nanoparticles

MgIONPs were fabricated by a thermal decomposition process in benzyl ether. Iron (III) acetylacetonate (0.7063 g), magnesium acetate tetrahydrate (0.02787 g), and oleic acid (0.3389 g) were mixed with benzyl ether (20 mL). The solution was transferred to a three-neck flask with a temperature sensor, nitrogen, a condenser, and a magnetic bar stirrer set on a heating mantel. The solution was then mixed well for 60 min at room temperature for degassing. The temperature was increased to 200 °C at a rate of 8 °C/min with vigorous stirring. Afterwards, the solution was maintained at 200 °C for 50 min. The temperature was then increased to 295 °C at a rate of heating of 5 °C/min with vigorous stirring and maintained at 295 °C for 60 min, before cooling to room temperature. The prepared NPs were washed twice with ethanol and separated using a magnet. 

#### 2.2.2. Surface Modification of MgIONPs with Tetramethyl Ammonium Hydroxide

To produce hydrophilic NPs for water dispersion, the prepared MgIONPs were coated with TMAH. In brief, 10 mL TMAH was dispersed in DI water (10 mL). The TMAH solution was then added to the prepared MgIONPs in an ultrasonic bath and further mixed for 30 min. The coated NPs were separated using a magnetic bar, washed with water, and then dried in an oven at 80 °C for 2 days. The obtained NPs powder was well dispersed in water and used for further studies.

### 2.3. Characterization

X-ray diffraction (XRD) of the NPs was carried out using an X-ray diffractometer (Rigaku, Tokyo, Japan). The crystallite sizes (*D*_p_) of the NPs were calculated using Scherrer’s formula, as follows [18]:

Crystallite size (*D*_p_) = Kλ/(*B*cos*θ*), (1)

*D_p_*: the average crystallite size (nm), *B*: the full width at half maximum of the XRD peak, *λ*: the X-ray wavelength (1.5406 Å), *K*: Scherrer’s constant (shape parameter: 0.89), and *θ*: the XRD peak position.

(2) $$\frac{1}{d_{hkl}^{2}}=\frac{h^{2}+k^{2}+l^{2}}{a^{2}}$$

The zeta potential and particle size analyzer (ELSZ-2000; Photal Otsuka Electronics Co., Osaka, Japan) was used to evaluate the absorbance properties using ultraviolet-visible spectroscopy. The magnetic behaviors of NPs were investigated using vibrating sample magnetometry (VSM; Lake Shore 7400 series; Lake Shore Cryotronics, Inc., Westerville, OH, USA). ICP-OES (Optima 8300, Perkin Elmer, Waltham, MA, USA) was performed to determine the metal content of the NPs. The morphology of NPs was examined by TEM (Tecnai G2S Twin; Philips, USA) at 300 keV. For the magnetic particle imaging (MPI) experiment, a gradient field of 2.6 T/m was applied to generate the field-free point, and an excitation field of 200 μT at 25 kHz was used to magnetize the particles.

Heating profile was evaluated using a custom-designed laboratory system. A function generator was used to generate a sinusoidal voltage signal, which was further amplified to the desired power through an alternating current power amplifier (AE Techron 7224, Elkhart, IN, USA). The SLP value was calculated using the following equation. Note that we used data obtained from the first 10 s.

*SLP* = (*C_p_/m*) × (*dT/dt*) (3)

*dT/dt:* time dependent temperature, *Cp:* 4.184 for water, and *m:* the mass of elements per volume. 

### 2.4. In Vitro Cytocompatibility Test

Cytocompatibility of the nanoparticles was evaluated through in vitro cytotoxicity tests with murine NIH-3T3 fibroblasts cells. First, the cytotoxicity of the nanoparticles was tested with a WST assay [19], which can quantitatively measure the metabolic activity of mitochondria in live cells. The culture medium (1 mL) containing the different NP contents (0.125, 0.25, 0.5, 1, or 2 mg/mL) was added to the cells and incubated for 24 h. Then, the cells were washed with sterile Dulbecco’s phosphate-buffered saline. Finally, fresh culture medium (0.5 mL) and WST assay solution (0.05 mL) were added to each well and incubated for an additional 2 h. Then, the absorbance of each sample solution at 450 nm was measured using a plate reader. We reported cell viability using the following equation:

*Cell Viability* (%) = (*A_c_* − *A_s_)/A_c_* × 100, (4)

*A_c_*: the absorbance of the control sample and *As:* the absorbance of the sample solution.

In addition, we examine the cytocompatibility of nanoparticles by using a Live/Dead staining kit (Invitrogen, Carlsbad, CA, USA) according to the manufacturer’s protocol. This assay results in the staining of live cells and dead cells with green and red colors, respectively. The percentage of live cells in the total cells (live and dead cells) after the exposure to the nanoparticles can indicate cytocompatibility. Fluorescence images were acquired using a fluorescence microscope (DMI3000B; Leica, Wetzlar, Germany).

## 3. Results and Discussion

### 3.1. Synthesis and Characterization of MgIONPs@TMAH

**Figure 1:**
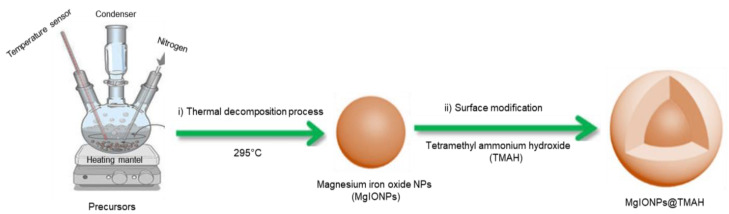
Schematic representation of the steps in the synthesis of MgIONPs@TMAH.

MgIONPs@TMAH were prepared and their efficiency as a theranostic agent for hyperthermia and MPI was investigated and compared with commercial iron oxide NPs. MgIONPs@TMAH were first prepared using a thermal decomposition process, followed by coating with TMAH to obtain relatively uniform water-dispersable NPs (Figure 1).

**Figure 2:**
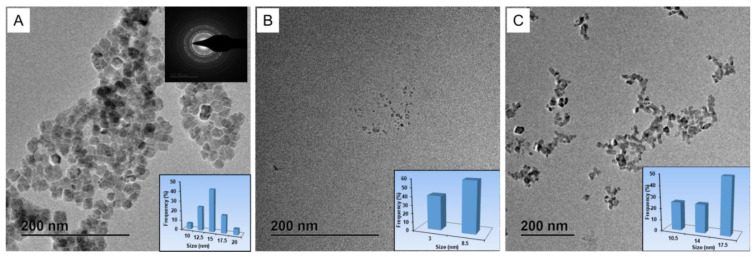
(**A**) TEM and SAED images of the prepared hydrophilic magnesium iron oxide@tetramethyl ammonium hydroxide nanoparticles (MgIONPs@TMAH), (**B**) RES NPs, and (**C**) BNF NPs.

The prepared MgIONPs@TMAH were relatively monodisperse, with a narrow size distribution (15.0 ± 5.0 nm) (Figure 2A). For comparison, we acquired TEM images of two commercial IONPs. RES NPs had an average particle size of 3.0–8.5 nm, whereas BNF NPs showed an average particle size (14.0 ± 3.5 nm) (Figure 2B,C). The crystalline nature of the prepared MgIONPs@TMAH was examined using high-resolution TEM/XRD; the results of which are discussed in the following section. The corresponding SAED image of the NPs displayed ring characteristics, which is proportional to a structure of small domains. The SAED pattern also showed widespread rings with a low intensity, indicating the reflection planes of the NPs. Generally, the presence of aggregation behavior between NPs is a result of high surface energy and magnetic dipole–dipole force. This detrimental aggregation was reduced by introducing a shell layer of polymer or organic material on the surface of the NPs [20,21].

**Figure 3:**
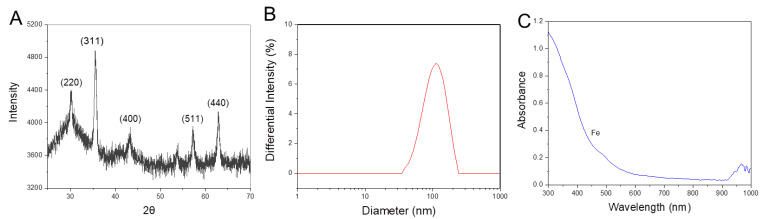
(**A**) XRD spectrum, (**B**) size distribution, and (**C**) absorbance spectrum of the prepared MgIONPs@TMAH.

Elemental compositions of the prepared MgIONPs@TMAH were quantified by ICP-OES. Element weight represented 58.52% of the entire NP content, in which Fe and Mg atoms were 58.3% and 0.22% in the composition, respectively. The crystalline properties of MgIONPs@TMAH were investigated and compared with the standard Joint Committee on Powder Diffraction Standards data (JCPDS: 88-1935) using XRD (Figure 3A) [22]. The indexed peaks were (220), (311), (400), (511), and (440), and magnesium ferrite structure was confirmed. The crystallite sizes and the associated lattice parameters for MgIONPs@TMAH were 13.89 nm and 8.38 Å, respectively. The crystalline properties of NPs were calculated based on maximum intensity peak. Peak broadening depends on various factors, such as instrumental effects, strain effects, and a finite crystallite size. The peaks were rather broad and weak, likely due to disorder and small crystallite effects. The crystalline properties of NPs are important for heating efficiency [23]. When the MNP size is maintained below a critical volume/size during the nanoparticle synthesis, the MNPs tend to behave as single magnetic domain structures, and at the smallest sizes, they exhibit superparamagnetic behavior under standard conditions. As results, the value of Ms increases with the size of the MNPs until it reaches a maximum that is close to the bulk magnetization value. As the size of the MNPs increases, they eventually possess pseudo single-domains and then multi-domain structures, in which the moment of each domain may not be oriented in the same direction [24]. As the relaxation time and magnetization of the NPs have an effect on the SLP value, a specific NPs size can exhibit effective heating [25]. Vreeland et al. recorded that approximately 22 nm is the effective size to enhance the SLP value for superparamagnetic NPs [25]. The diameter and shape of magnetic NPs are also important factors associated with heating efficiency. Core-shell NP cubes exhibit a high heating profile, due to low anisotropy and spin disorder reduction [23].

The mean hydrodynamic size of MgIONPs@TMAH obtained by DLS was 167.0 ± 0.8 nm (Figure 3B). Note that this value is slightly higher than that obtained from TEM analysis, which is typical for hydrophilic NPs due to water–material interactions.

The zeta potential (ζ) of the MgIONPs@TMAH was investigated, as it is closely related to the stability of nanoparticles. The magnetic NPs stability is significant in biomedical applications [26]. A low zeta potential value (−12.0 ± 0.1 mV) implies that the NP may show poor stability in aqueous solutions. ±30 mV is considered the limit value for setting stability in colloidal systems, due to the formation of a high repulsion force between nanoparticles at this value [27].

The absorbance of the prepared NPs was measured by UV-Vis spectroscopy. MgIONPs@TMAH spectra showed broad absorption over the visible range (300–600 nm), as a result of the presence of *d*-orbital in Fe^3+^ (Figure 3C). In particular, the absorption peak at 490 nm corresponded to Fe^3+^ in a tetrahedral coordination [28,29,30].

**Figure 4:**
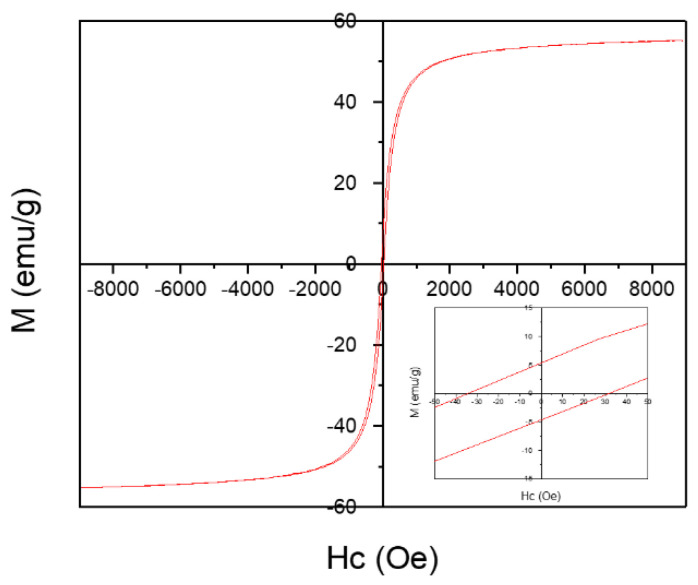
VSM of the prepared MgIONPs@TMAH.

The magnetic behavior of MgIONPs@TMAH was studied using VSM at room temperature (~23 °C). The magnetization (M)–field strength (H) curves showed hysteresis loops, indicating the ferromagnetism of the prepared MgIONPs@TMAH with a magnetization saturation (*M_s_*) at 55.1 emu/g (Figure 4). MgIONPs@TMAH showed very low coercivity (*Hc*) and a remanence magnetization (*M_r_*) of 30.2 Oe and 5.5 emu/g, respectively, due to the soft magnetic nature of the NPs. The squareness (SQ) is defined as the ratio of remanence magnetization to magnetization saturation. A single magnetic domain structure is observed when SQ ≥ 0.5, whereas a material having SQ < 0.5 is considered to have a multi-domain structure. In our study, MgIONPs@TMAH samples showed the presence of multi-domain structures [31]. Performing a ZFC-FC analysis is useful to obtain the blocking temperature, and thus the information about the superparamagnetic state at RT. However, unfortunately, ZFC-FC analysis is not available at the moment. So, it is a limitation for this research that will be performed as future work.

### 3.2. Hyperthermia Performance

SLP was used as an indicator to evaluate the absorbed energy amount per metal NP mass under the action of an AMF [2]. In general, the SLP (heat generation) of NPs under an external AMF depends on Néel and Brownian relaxations [3]. Importantly, for human exposure, it is pivotal to maintain the product of the magnetic field strength (*H*) and its frequency (*f*) below a threshold safety value known as the Brezovich criterion. Based on this safety limit, the product of the frequency and the field amplitude (C = *H × f*) should remain below 5 × 10^9^ A m^−1^s^−1^ to minimize any collateral effects of alternating magnetic fields on the human body [2,3]. Heating efficiency of the prepared nanoparticles was investigated by applying AC magnetic fields of various strengths (40 or 50 kA/m) at a frequency of 97 kHz. The values of C in our experiments were calculated to be 3.8 × 10^9^ and 4.8 × 10^9^ A m^−1^s^−1^ for 40 and 50 kA/m, respectively, which did not exceed the safety limit. On the other hand, at a constant field strength of 13.5 kA/m, while varying the frequency from 159.8 to 269.9 kHz, the values of C in our experiments were calculated to be 2.1 × 10^9^ and 3.6 × 10^9^ A m^−1^s^−1^ for 159.8 and 269.9 kHz, respectively. These values also did not exceed the safety limit. However, in the case of using the low field strength of 9.5 kA/m and a high field frequency of 614.4 kHz, the value of C in our experiments was calculated to be 5.8 × 10^9^ A m^−1^s^−1^, which slightly exceeds the safety limit. For the hyperthermia system in this study, the SLP values were obtained by employing the effects of field frequency and field variation to investigate and optimize the heating effects.

#### 3.2.1. Effects of Magnetic-Field Strength Variation with a Constant Frequency

**Figure 5:**
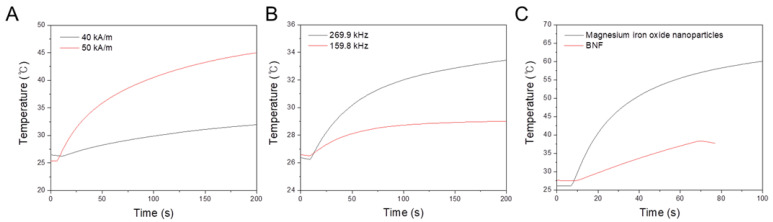
Temperature elevation by (**A**) MgIONPs@TMAH in response to the application of AMF with a constant frequency of 97 kHz, while varying the strength from 40 to 50 kA/m. (**B**) MgIONPs@TMAH in response to the application of an AMF with a constant strength of 13.5 kA/m, while varying the frequency from 159.8 to 269.9 kHz. (**C**) MgIONPs@TMAH (36.5 mg/mL) and BNF NPs (60 mg/mL) with the application of an AMF having a frequency of 614.4 kHz and a strength of 9.5 kA/m.

The heating efficiency of MgIONPs@TMAH was investigated at a constant frequency of 97 kHz, while varying the strength (40–50 kA/m), as shown in Figure 5A. The increase in temperature against time was mostly linear, and its rate gradually slowed. The rate of heating increased with the strength (Figure 5A). Accordingly, the heating efficiency increased as the strength increased from 40 to 50 kA/m. For example, the increases in temperature were 6 and 20 °C after 200 s of AMF applications of 40 and 50 kA/m, respectively.

#### 3.2.2. Effects of Field Frequency Variation with a Constant Magnetic Field Strength 

The heating efficiency of MgIONPs@TMAH was investigated at constant field strength of 13.5 kA/m, while varying the frequency from 159.8 to 269.9 kHz, as shown in Figure 5B. The heating efficiency increased with the variation of field frequency from 159.8 to 269.9 kHz. As a result, the temperature elevations were 2.5 °C and 7 °C after 200 s of AMF applications at 159.8 and 269.9 kHz, respectively.

#### 3.2.3. Effects of a High-Field Frequency with a Low Magnetic Field Strength

The heating efficiency of MgIONPs@TMAH was investigated and compared with BNF under the conditions of low field strength of 9.5 kA/m and a high field frequency of 614.4 kHz, as shown in Figure 5C. During the first 70 s of AMF application, a temperature change of 26 °C was observed for the prepared NPs, whereas a temperature change of only 10.5 °C was observed for BNF. This indicates that the heating profile of the MgIONPs@TMAH is substantially better than that of the commercial NPs.

Under the aforementioned conditions, the heating profile of the fabricated NPs was enhanced under the field of frequency 614.4 kHz and strength 9.5 kA/m. Comparisons of the SLP and intrinsic loss parameter (ILP) of the MgIONPs@TMAH and the commercial NPs are shown in Table 1. The difference in ILP is high, which indicates that the sample is dependent on applied field and agglomerating, which is probably caused by the low stability of the suspension. 

We successfully obtained magnetic NPs exhibiting a high SLP under specific conditions with the given magnetic field parameters and a saturation temperature of 45 °C, suggesting that MgIONPs@TMAH can be used in cancer treatment. The heating time needed for the temperature to reach 45 °C is affected by various factors, including magnetic field conditions, the viscous medium, and the diameter and sample concentration of the magnetic NPs. Localized hyperthermia using NPs under low magnetic fields is favorable for treating small or deeply set tumors. It was reported that zinc-doped magnetite cubic nanoparticles show a 5-fold improvement in the specific absorption rate (SAR) in magnetic hyperthermia while providing a good MPI signal, when comparing with un-doped spherical nanoparticles [5].

### 3.3. Magnetic Particle Imaging Performance

It is notable that, due to the exploitation of magnetization, the tracer used in MPI is similar to that used in magnetic hyperthermia. The mechanism of heat generation in magnetic hyperthermia depends on the Brownian and Néel relaxation principles for magnetization reversal, which is similar to the signal generation mechanism in MPI. To generate a signal, MPI tracers must undergo magnetization reversal, which requires the overcoming of inertial torques and viscous and thermal effects using the Néel and Brownian relaxations. Modulation of the diameter and particle shape of NPs can tune the magnetic behavior, induce saturation magnetization, and further enhance magnetic hyperthermia properties and MPI [5,6,7]. SLP and the spatial resolution of MPI are influenced by the saturation magnetization, as shown by Equation (5) for SLP and Equation (6) for MPI:

(5) $$SLP=\frac{\pi \cdot \mu_{0}\cdot \chi^{\prime \prime }\left(f\right)\cdot H^{2}\cdot f}{\rho_{\mathrm{MNPs}}\cdot \phi },\;\mathrm{where}\;\chi^{\prime \prime }\left(f\right)=\frac{\mu_{0}M_{s}^{2}V}{3k_{B}T}\frac{\omega \tau_{R}}{\left(1+\omega^{2}\tau^{2}{}_{R}\right)},$$

*f* and *H*: the frequency and magnetic field strength, *ρ*_MNPs_: the magnetic NP density, *ϕ*: the magnetic NP volume fraction, *µ*_0_: the permeability, and *χ*”: the susceptibility. *M*_s_: the magnetization, *V*: the volume of the magnetic NP, ω = 2π*f*: the AMF sweep rate, *τ_R_*: the relaxation time, and *k*_B_: Boltzmann’s constant.

(6) $$\mathrm{MPI}:\;\mathrm{spatial}\;\mathrm{resolution}\approx \frac{24k_{B}T}{\mu_{0}\pi M_{s}}G^{-1}d^{-3},$$

*G:* selection field gradient, *d*: magnetic diameter NP, *T*: temperature of the magnetic NP.

**Figure 6:**
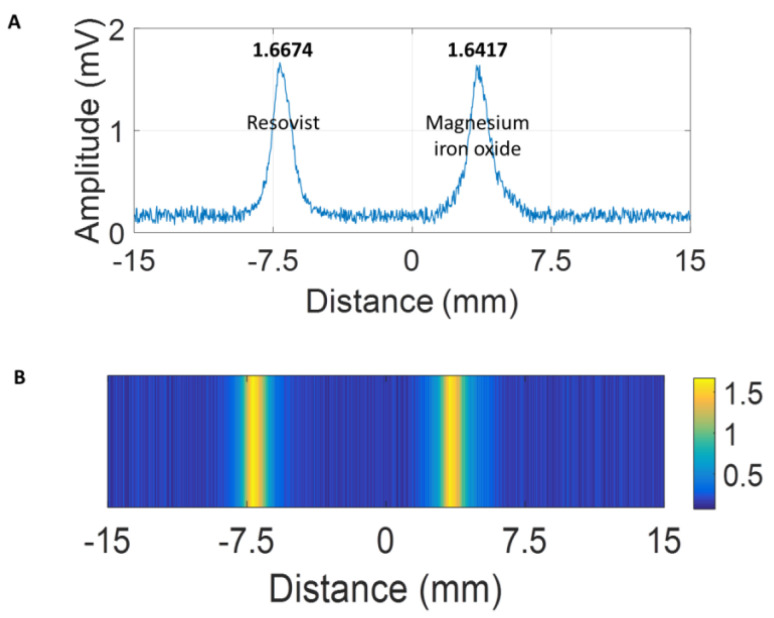
(**A**) Signals and (**B**) magnetic particle imaging (MPI) images of RES NPs and MgIONPs@TMAH.

MPI allows for the monitoring of the locations and distributions of magnetic NPs. The performance of the MgIONPs@TMAH (36.5 mg/mL) as a contrast agent for MPI was compared with that of RES NPs (36.5 mg/mL). RES NPs (100 µL) and MgIONPs@TMAH (100 µL) were subjected to an excitation field to generate the MPI signals, of which images were acquired from two positions using 1D-MPI, as shown in Figure 6B. MgIONPs@TMAH produced a signal approximately the same as that of RES NPs, as shown in Figure 6A; thus, the imaging capability of MgIONPs@TMAH was recognized by 1D-MPI.

### 3.4. In Vitro Cytotoxicity Tests

**Figure 7:**
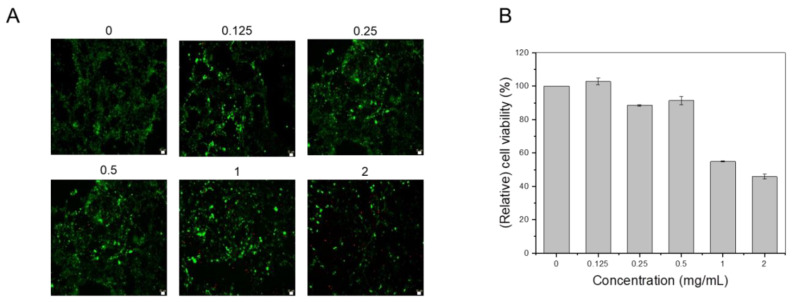
(**A**) Live/dead fluorescence imaging of the cultured cells and (**B**) cytocompatibility examined using a WST assay for various NP concentrations.

The cytotoxicity of MgIONPs@TMAH was examined with NIH-3T3 fibroblasts as shown in (Figure 7). For in vitro cytotoxicity tests, we performed both metabolic activity measurements using a WST assay (Figure 7B) and live/dead staining (Figure 7A). A WST assay can indicate cell viability based on colorimetric products by measuring the absorbance of the medium. It is important to note that there can be a variation in measurement (and error), which sometimes results in values over 100% (higher absorbance than the control). The relative cell viability of the sample (0.125 mg/mL) was not significantly different from that of the control sample. The cytotoxicity of the MgIONPs@TMAH showed dose dependence. The viability of the cells somewhat decreased with the increasing concentrations (1 and 2 mg/mL). WST results were in agreement with those of live/dead staining (Figure 7A). With 0–0.5 mg/mL of MgIONPs@TMAH, most of the cells were stained live, with only a small number of red-stained (dead) cells. These results indicate that the MgIONPs@TMAH have low cytotoxicity (<1 mg/mL).

The major issue in the developed nanomaterial is the high tendency of particles to agglomerate, even after the functionalization. It is fundamentally one of the most important factors for biomedical applications and still a challenging target [32,33,34,35,36]. Magnetic nanoparticles have large surface energy that causes the colloidal instability and aggregation. To mitigate this issue, layering of various coating materials, such as surfactants, has been commonly performed either during or after the synthesis of the nanoparticles. In general, post synthesis techniques can be introduced to modify nanoparticles to avoid aggregation or to stimulate disaggregation. The formation of a layer on the particle can prevent aggregation. If the synthetic route allows for the inserting of surface coating molecules before aggregation takes place, the particles can remain well dispersible in target media straight away. Additionally, surface molecules can be exchanged through chemical synthesis after the particle synthesis. Such post synthetic technique offers the possibility for a variety of surface modifications, which can be adjusted to the required applications. In some cases, especially in water, stability of nanoparticles is strongly affected by their surface charge. This behavior is influenced by the ion content of the water and the amount of charged ions on the surface of the nanoparticles. Surface modification and surface charge can have a major impact on the biological response to particles, including phagocytosis and genotoxicity; hence, these parameters need to be controlled. However, if the surface properties cannot be controlled, nanoparticles quickly form large particles due to agglomeration. Also, the heating efficiency of nanoparticles is affected by the size of the nanoparticles and the coating layer. Hence, the magnetic nanoparticles should be prepared with a proper method to reach the target temperature [37,38].

The average particle diameter in the single domain (from 20 to 70 nm) along with a narrow size distribution was reported to exhibit good heating performance [39]. The reported values for SLP in the literature are in the range of 10–100 W/g for magnetic field conditions of *H* = 10 kA/m and *f* ≈ 400 kHz [40]. Nigam et al. reported that magnetite nanoparticles coated with citrate had a *Ms* value of 57 emu/g with high SLP to reach the target temperature (43 °C) in a short time [41]. High SLP (150 W/g) was reached by Reyes-Ortega et al. under a magnetic field condition of *H* = 20 mT and *f* = 205 kHz [42]. Magnetite nanoparticles stabilized with chitosan (15 nm) showed higher SLP (119 W/g) than uncoated magnetite nanoparticles likely due to the enhancement in the particle dispersion, which was obtained by the coating of the hydrophilic shell layer [43]. The magnetic fluid MFL AS (Nanotherm^®^, MagForce Nanotechnologies AG, Berlin, Germany), which consists of superparamagnetic iron oxide nanoparticles dispersed in water at an iron concentration of 112 mg/mL, has been approved for clinical trials. The iron oxide core is covered by an aminosilane-type shell and its size is approximately 15 nm in diameter. The required magnetic field to reach the target temperature with a frequency of 100 kHz and a variable field strength of 0–18 kA/m is generated in the MFH^®^300F applicator. We compared the results of this study to those reported in the literature, as summarized in Table 2.

## 4. Conclusions

In summary, we have successfully reported the synthesis of hydrophilic MgIONPs@TMAH through a two-step process consisting of thermal decomposition and the subsequent coating with TMAH to obtain relatively uniform water-dispersable NPs (15.0 ± 5.0 nm). For the hyperthermia system, the heating efficiencies were investigated and optimized by differing field frequency and field strength. The heating efficiency of the prepared nanoparticles was enhanced significantly more by the increase in the field strength (from 40 to 50 kA/m) than by the increase in the field frequency (from 159.8 to 269.9 kHz). Under magnetic field conditions of frequency 614.4 kHz and strength 9.5 kA/m, MgIONPs@TMAH nanoparticles show a 15-fold improvement in the specific loss power (SLP) in magnetic hyperthermia as compared to the BNF nanoparticles. MgIONPs@TMAH produced an MPI signal that was approximately the same as that produced by Resovist nanoparticles when subjected to an excitation field. The imaging capability of the prepared nanoparticles was recognized by 1D-MPI. We successfully obtained magnetic nanoparticles that exhibit a high SLP under specific conditions with the given magnetic field parameters, a saturation temperature of 45 °C and a good MPI signal, suggesting that the MgIONPs@TMAH can be useful for various biomedical applications, such as image-guided cancer treatment.

## Figures and Tables

**Table 1:** Comparisons of the SLP and ILP of the prepared NPs and the commercial NPs (BNF).

Conditions	*f* = 614.4 kHz; *H =* 9.5 kA/m	*f* = 97 kHz; *H* = 40 kA/m
MgIONPs@TMAH	SLP: 216.18 W/g ILP:3.8 nHm^2^/kg	SLP:10.84 W/g ILP:0.069 nHm^2^/kg
BNF	SLP:14 W/g ILP:0.25 nHm^2^/kg	SLP:195.2 W/g ILP:1.25 nHm^2^/kg

**Table 2:** Comparison between particles reported in the literature and the current study.

Sample	Size (nm)	M_s_ (emu/g)	SLP	Alternating Current (AC) Field Condition	ILP	MPI Good Performance
Chitosan-coated Fe_3_O_4_ [44]	37	71.5	595	*H*: 14, *f*: 335	9	-
PEG-coated Fe_3_O_4_ [45]	31	54	355	*H*: 27, *f*: 400	1.22	-
Fe_3_O_4_ [46]	37	67	213	*H*: 23.9, *f*: 571	0.72	-
Fe_3_O_4_,Sph [5]	19.2 ± 1.3	101.5	189.6	*H*: 16, *f*: 380	1.9	+
Fe_3_O_4_,Cube	15.5 ± 1.1	107.3	356.2		3.6	
Zn_0_._4_Fe_2_._6_O_4_,Zn-Sph	19.1 ± 1.0	125.7	438.6		4.5	
Zn_0_._4_Fe_2_._6_O_4_,Zn-Cube	15.4 ± 1.1	130.4	1019.2		10.47	
FeCo@C [7]	40 nm	192	406	*H*: 100, *f*: 30	1.3	+
Citrate-coated IONPs [6]	10.6 ± 1.8	73.8	230	*H*: 46, *f*: 186	0.58	+
	13.1 ± 2.2	82.5	350		0.88	
Fe_3_O_4_@ZnO [47]	10	31.2	80	*H*: 25.12, *f*: 250	0.49	-
MgIONPs@TMAH	15.0 ± 5.0	55.1	216.18	*H*: 9.5, *f*: 614.4	3.8	Current study

## Data Availability

The data is contained within the article.
